# Comparison of Low-Dose Promethazine and Dexamethasone against Ondansetron Monotherapy Given as Antiemetic Prophylaxis during Myomectomy Under Spinal Anesthesia: A Randomized Clinical Trial

**DOI:** 10.1155/2022/2094662

**Published:** 2022-08-25

**Authors:** Emmanuel Onokpite, Abiodun Oyinpreye Jasper, Philomina Nosa Edomwonyi

**Affiliations:** ^1^Department of Anaesthesia, Delta State University Teaching Hospital, Oghara, Delta, Nigeria; ^2^Department of Anesthesiology, University of Benin Teaching Hospital, Benin, Nigeria

## Abstract

**Background:**

Postoperative nausea and vomiting (PONV) prophylaxis is still inadequate for a significant proportion of women undergoing myomectomy under spinal anesthesia; and it substantially decreases patient's quality of postoperative recovery. Current protocol and practice favor the use of combination therapy like promethazine/dexamethasone for PONV prophylaxis with minimal side effects and cost advantages in low-resource climes. *Methodology*. Seventy American Society of Anesthesiologist (ASA) class I or II women aged 21–65 years scheduled for myomectomy were recruited and randomized into group A (promethazine/dexamethasone group) and group B (ondansetron group). Myomectomy was performed on each patient using spinal anesthesia. After induction of spinal anesthesia, patients in group A received intravenous promethazine 12.5 mg and dexamethasone 8 mg while group B received intravenous ondansetron 8 mg. Early (0–3 h) and late (4–24 h) PONV was assessed using the numerical scoring scale.

**Results:**

Data analysis was done using SPSS version 20. Postoperatively, there was no significant difference in the incidence of early ansd late PONV (*p* value >0.05) despite the higher incidents in the ondansetron group. The proportion of patients who required rescue antiemetics was more in the ondansetron group when compared with the promethazine/dexamethasone, with minimal and statistically insignificant side effects in both groups. There was significant patient satisfaction in both groups.

**Conclusion:**

The study shows that the combination of low-dose promethazine and dexamethasone is comparable to ondansetron when used as prophylaxis for PONV with cost benefits in low-resource environments.

## 1. Introduction

Postoperative nausea and vomiting (PONV) are one of the common complications following surgery and anesthesia. [1] When associated with retching, it may cause esophageal tear, surgical wound disruption, and delayed discharge from the postanesthetic care unit. The incidence of postoperative nausea and vomiting is about 30% in the general population and up to 60–70% in some high-risk patients. [1] The risk factors for PONV include the history of PONV, female gender, motion sickness, nonsmokers, perioperative use of opioids, and gonadotropins. In a study carried out by Tobi et al., [2] the incidence of postoperative nausea and vomiting was found to be 33.3% following myomectomy under spinal anesthesia.

There is presently no single drug with satisfactory control of PONV; further worsened by hypotension post-sympathectomy with spinal anesthesia. [3] Promethazine is a first-generation phenothiazine derivative with antihistamine, anticholinergic, and antidopaminergic blocking effects, with a duration of action of 4–6 h. Since its introduction in 1946, it has been used for the prevention and treatment of nausea and vomiting caused by narcotic therapy, migraine episodes, cancer chemotherapy, and allergic reactions. [4] It is a cheap and readily available antiemetic drug with its dominant side effect being sedation.

Dexamethasone (a synthetic steroid) with anti-inflammatory properties, is now increasingly used as an antiemetic in surgical patients and patients on chemotherapy. [5] In addition, dexamethasone was also found to reduce the incidence of late PONV; and have minimal effect on early PONV in patients undergoing myomectomy under spinal anesthesia. [2] This action is probably mediated via inhibition of either prostaglandin synthesis or the release of indigenous opioids. [6] Its major drawback of worsening infection and delayed wound healing limits its extended use in patients. [6–8]

The introduction of ondansetron in 1991 heralded, is a major advancement in the treatment of PONV. The drug is not associated with adverse effects that were observed with commonly used antiemetic drugs. [9] Ondansetron produces no sedation, no extrapyramidal symptoms, or adverse effects on vital organs. [9] However, it is costly.

A combination of drugs that act on different receptor sites, with a better therapeutic value, affordability, and availability becomes imperative. There is a paucity of reports on the use of low-dose promethazine as a prophylactic antiemetic in the West African subregion.

### 1.1. Objective

This study aims to evaluate the effectiveness of combination therapy of low-dose promethazine and dexamethasone against ondansetron monotherapy in the prophylaxis of PONV in a randomized clinical trial. The null hypothesis is that there is no difference between the combination therapy of low-dose promethazine/dexamethasone compared with monotherapy of ondansetron.

## 2. Method

This was a prospective, randomized double-blind, clinical study conducted in a tertiary institution. The study population was drawn from ASA 1 or 2 female patients aged between 21 and 65 years scheduled for myomectomy under spinal anesthesia after informed consent. Aside from outright refusal, patients with a history of PONV, history of motion sickness, 24 h history of steroids and intestinal obstruction, cardiovascular disease, or neurologic disorders were excluded from the study. Others excluded were patients with psychiatric illness, allergy to the study drugs, and history of low back pain or spinal column surgery.

They were educated on the numerical scoring system (NSS) for the assessment of PONV.

Ethical clearance was obtained from the Ethics and Research Committee of the Hospital. Routine preoperative assessment and laboratory work-up (urinalysis, full blood count, urea, and electrolytes) were done. All patients were fasted for at least 6–8 h for solid food and at least 2 h for clear fluids before the procedure. Each patient was premedicated with diazepam 5–10 mg orally, the night (10 pm) before and on the morning of the surgery.

A total of 70 were recruited. Baseline vital signs were done using a multiparameter monitor. After securing an intravenous line using a 16G cannula, the patient's circulatory system was preloaded with 15 ml/kg lactated Ringer's solution warmed to body temperature. Continuous monitoring of ECG, NIBP, HR, and SpO_2_ was done throughout the period of the study preop, intraop, and post op. Using a double-blind technique, patients were categorized into group A (*n* = 35) and group B (*n* = 35). Group A had promethazine 12.5 mg and dexamethasone 8 mg while group B had 8 mg ondansetron intravenously after the administration of subarachnoid block 10–15 mg (2–3 ml) of 0.5% hyperbaric bupivacaine plus 15–20 mcg of fentanyl was deposited into the subarachnoid space.

Fluid maintenance was achieved with Ringer's lactate 10 ml/kg for the first 1 h and then 5 ml/kg subsequently, blood transfusion commenced if indicated. The sensory block level was assessed by a gentle pinprick with a short bevel needle and the desired sensory block was T_4_–T_6_. Motor block was assessed using the Bromage scale. Intraoperative nausea and vomiting were treated with 10 mg metoclopramide.

The incidence of PONV was monitored for the first 3 h (early PONV) and the next 21 h (late PONV) postoperatively. Vital signs such as pulse rate, blood pressure, SpO_2_, and temperature were continuously monitored every 10 min in the recovery room until discharge to the ward. Vital signs monitoring continued in the ward until the end of the first 24 h after surgery. Patients were questioned about the incidence and severity of PONV in the ward during the postoperative visits The severity of PONV was graded as 0 = no nausea or vomiting, 1 = nausea, no vomiting, 2 = vomiting once, and 3 = 2 or more episodes of vomiting. Any untoward side effects such as nausea, vomiting, respiratory depression, sedation, shivering, bradycardia, or hypotension were recorded.

Hypotension (BP < 90/60 mmHg) was treated with intravenous fluid boluses, or 3 mg of ephedrine in aliquots. Bradycardia (HR < 60 BPM) was treated with iv atropine 0.01 mg/kg. Patients observed to have sedation were given oxygen via a face mask at 8 l/min, shivering was treated with warm fluids, use of a space blanket, and Bair hugger, as well as oxygen via nasal prongs.

The level of sedation was assessed using the Ramsay sedation score. Sedation was regarded as present, if the score is > 3. Early PONV was 0–3 h while late PONV was the period from the 4th hour to the 24th hour postoperatively. Data form was used to document demographic characteristics, baseline vital signs, intraoperative events, side effects, postoperative complications, management, and outcomes.

The primary outcome was the measurement of the incidence of early and late PONV. The secondary outcome was the incidence of side effects and the determination of patient's satisfaction with antiemetic prophylaxis using a 5-point Likert scale of 1 to 5 corresponding to very poor, poor, good, very good, and excellent.

Data were collected prospectively using the attached proforma. Statistical analysis using SPSS version 20. Simple independent student's *t-*test (2-tailed) was used to analyze continuous patient's variables like age, weight, duration of surgery, and anesthesia. Chi-square and Fisher's exact tests were used for discrete variables like symptoms of PONV. A *p* value of <0.05 was taken as being significant.

## 3. Results

A total of 70 patients of the American Society of Anesthesiologists (ASA) Class 1 or Class 2, aged between 21 and 65 years took part in the study: 35 patients in group A (promethazine/dexamethasone group) and 35 patients in group B (ondansetron group). Data obtained from 70 patients were analyzed.  Table1 shows the demographic and clinical characteristics of the patients which did not differ significantly between the two groups (*p*-value = 0.532, 0.336, 0.738, 0.235, and 0.259, respectively).

Baseline vital signs are shown in Table 2. The pulse rate (*p*-value=0.238), systolic blood pressure (*p*-value=0.320), diastolic blood pressure (*p*-value=0.268), mean arterial pressure (*p*-value=0.065), respiratory rate (*p*-value=0.496), and oxygen saturation (*p*-value=0.489) did not differ significantly between the two groups.

**Figure 1:**
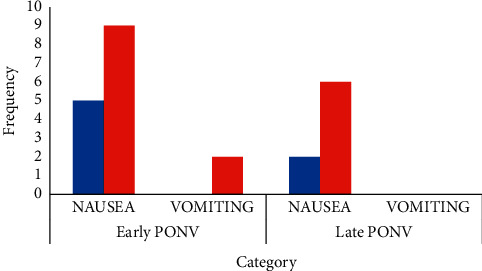
The incidence of nausea and vomiting.

**Figure 2:**
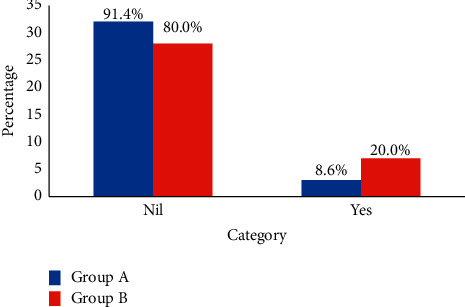
Proportion of patients given rescue antiemetic.

The incidence of early and late PONV are shown in Table 3. The overall incidence of PONV was 34.3%. A total of 5 (14.3%) patients in the promethazine/dexamethasone group and 11 (31.4%) patients in the ondansetron group had early PONV, while 2 (5.7%) patients in the promethazine/dexamethasone group and 6 (17.1%) patients in the ondansetron group had late PONV. However, these differences did not reach statistical significance (*p*=0.149 and 0.259, respectively).  Figure 1 reveals the incidence of PONV. While 5 (14.3%) patients in the promethazine/dexamethasone group had nausea in the early period compared with 9 (25.7%) patients in the ondansetron group, none in the promethazine/dexamethasone group had vomiting in the early period compared to 2 (5.7%) patients in the ondansetron group. Moreover, 2 (5.7%) patients in the promethazine/dexamethasone group had nausea in the late period compared with 6 (17.1%) patients in the ondansetron group. However, no patient in both groups had vomiting in the late period. As seen in Figure 2, three (8.6%) patients in the promethazine/dexamethasone group received rescue antiemetics compared with seven (20.0%) patients in the ondansetron group, but the difference was not statistically significant (*p*=0.306).

**Figure 3:**
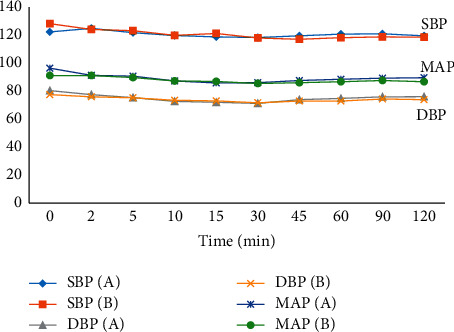
Trends of hemodynamic parameters (systolic, diastolic, and mean arterial blood pressure).

**Figure 4:**
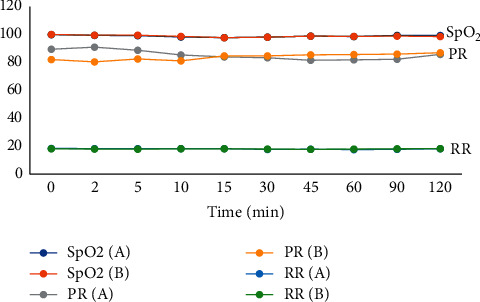
Trends of pulse rate (PR), respiratory rate (RR), and peripheral oxygen saturation (SpO_2_).

Intraoperative block characteristics such as the degree of motor block and dermatomal sensory block height are shown in Table 4. No significant difference was found in the degree of motor block (*p*=0.355) or dermatomal sensory block height (*p*=0.641).  Figure 3 shows the intraoperative variations of systolic blood pressure, diastolic blood pressure, and mean arterial pressure (MAP), while Figure 4 shows the intraoperative variations of the pulse rate, respiratory rate, and oxygen saturation (SpO_2_). There was no statistically significant difference between the two groups in terms of hemodynamic variables during the period under observation.

Intraoperative and postoperative complications are shown in Table 5. Hypotension was observed equally in both groups. Bradycardia was observed in five patients, four in the promethazine/dexamethasone group and one in the ondansetron group. However, the difference did not reach statistical significance (0.325). Sedation occurred in seven patients in the promethazine/dexamethasone group compared to with patients in the ondansetron group, but the difference did not reach statistical significance (*p*=0.324). Few patients experienced shivering, pruritus, and respiratory depression in both groups, but the differences were not statistically significant (*p*-value=0.752, 1.000, and 1.000, respectively). No patient experienced PDPH and urine retention. However, two patients experienced backache in group A and one patient in group B, but it was not statistically significant, *p*=1.000.

There was no statistically significant difference in the patient's satisfaction with antiemetic prophylaxis between the two groups as shown in Table 5 (*p*-value=0.705). The majority of patients rated their satisfaction as very good (42.9% vs. 45.7%) or excellent (42.9% vs. 34.3%) in the promethazine/dexamethasone group and the ondansetron group, respectively.

## 4. Discussion

The overall incidence of PONV in this study was 34.3% (Table 3), despite the comparable demographics and subarachnoid block characteristics in all the patients (Tables 1–3). This is comparable to the incidences of 33.3%, 29.5%, 33.6%, 36.5%, and 38.4%, respectively, in earlier studies. [2] However, some other studies reported a comparatively higher incidence of 55.3%, 46.7%, and 51.6%, respectively. [10] An overall high incidence of postoperative nausea and vomiting of 69.5% was reported by Talebpour et al., [11] following the administration of a combination of promethazine/dexamethasone and metoclopramide/dexamethasone for bariatric procedures: known to predispose to PONV. [11] Though Gan et al. [12] used promethazine alone resulting high incidence of PONV, our results showed otherwise with the promethazine/dexamethasone combination.

The overall incidence of PONV in the promethazine/dexamethasone group was 20% (Table 3). This is comparable with 20.6% and 23% reported by Jan et al. [13] and Daabiss et al. [14] However, Bergess et al. [15] reported a higher incidence of 31.0% and 36.2% in both arms of their study, while Talebpour et al. [11] and Habib et al. [16] reported 41% and 30% incidence, respectively, in their study despite a triple therapy used in the Bergess et al. [15] study involving aprepitant, promethazine, and dexamethasone combination in one group and ondansetron, promethazine, and dexamethasone combination in the other group, the patients still experienced a higher incidence of PONV compared with the patients in our promethazine/dexamethasone group. Moreover, in the Talebpour et al. [11] study, promethazine was given intramuscularly while dexamethasone was introduced after 24 h. We gave a combination of promethazine and dexamethasone intravenously in the first 24 h. Meanwhile, Gan et al. [12] carried out their study in women undergoing gynecological laparoscopic procedures. Though females are known to have higher risks of PONV, combination therapy of low dose promethazine (6.25mg) and granisetron yielded a lower incidence of PONV against a higher dose of 12.5mg promethazine monotherapy. In the same vein, dexamethasone acted synergistically to potentiate the effects of low dose promethazine in our study In addition, dexamethasone may have acted synergistically to potentiate the effects of promethazine.

Conversely, Jan et al. [15] and Singer et al. [17] reported a much lower incidence of PONV of 18.9% and 11.5%, respectively, compared with the 20.0% reported in promethazine/dexamethasone. Jan et al. [13] administered a high dose of metoclopramide (50 mg) combined with 8 mg dexamethasone, which led to a very low incidence of PONV in one of their study groups. Likewise, Singer et al. [17] carried out a pilot study in a pediatric population where a relatively high dose of dexamethasone (10 mg) was administered.

Furthermore, Shirdashtzadeh et al., [18] Singer et al., [17] and Kumar et al. [19] in their studies recorded a lower incidence of 0%, 0%, and 10.0%, respectively, compared with the 20.0% recorded in the index study in the promethazine/dexamethasone group. In the study by Shirdashtzadeh et al., [18] patients for appendectomy received a much higher dose of promethazine (1 mg/kg) compared with 12.5 mg that we used in these patients for myomectomy, which is a risk factor for PONV. Singer and colleagues [17] administered initial and follow-up doses of the study drugs postoperatively. This contrasts with the index study where a single dose of the study drugs was given prior to surgery. The cumulative effects of the follow-up doses may have resulted in the higher efficacy and lower incidence of PONV as seen in Singer et al.'s study. In the study by Kumar et al., [19] the authors combined ondansetron and dexamethasone for prophylaxis of PONV. The differences in combination by these authors may be responsible for the differences in outcomes.

The overall incidence of PONV in the ondansetron group in the index study was 48.6% (Table 3). This is comparable to the incidences of 49%, 49%, and 44% reported by Szarvas et al., [10] and Kim et al., [20] respectively. However, in other studies where patients had general anesthesia, lower incidences of 13.4%, 14%, and 35% were reported, respectively. [19, 21, 22] General anesthesia using volatile anesthetics is associated with an average incidence of PONV ranging between 20% and 30%, particularly when using anesthetics such as isoflurane in combination with propofol. [23, 24] This may be responsible for the lower incidence reported by these authors. Moreover, Chakraborty et al. [22] did not include retching as part of their PONV parameters.

In contrast, Ommid et al., [25] Samieirad et al., [26] and Cruz et al. [25] reported a higher incidence of 52%, 53.3%, and 62%, respectively. Ommid et al. [25] conducted their study on female patients undergoing laparoscopic cholecystectomy under general anesthesia. Female carries a very high risk for the development of PONV, especially when undergoing laparoscopic surgeries like cholecystectomy, with an incidence as high as 72%. [27] This may explain why Ommid and colleagues observed a higher incidence of PONV in their study. Samieirad et al. [26] administered ondansetron orally unlike the index study where it was administered intravenously. The bioavailability of ondansetron after oral administration is about 56%. The reduced bioavailability from the oral route may have resulted in the higher incidence of PONV reported by the authors. [28] Cruz et al. [24] used 4 mg of ondansetron in their study as with the 8 mg that we used. The reduced dose of ondansetron (4 mg) by Cruz and colleagues may have reduced the effectiveness of ondansetron in their study.

The incidence of early PONV and late PONV was much lower in the promethazine/dexamethasone group compared with the ondansetron group (Table 3, Figure 1). This probably may be due to the multimodal mechanism of action of promethazine (antihistamine, anticholinergic, and antidopaminergic) in combination with dexamethasone. However, there was no statistically significant difference between the two groups regarding early PONV (*p*=0.149) or late PONV (*p*=0.259). Cruz et al. [24] compared ondansetron/dexamethasone combination with ondansetron alone and found no significant difference between the groups regarding early PONV, but there was a significant difference between the groups regarding late PONV. Similarly, Rajeeva et al. [29] found that ondansetron/dexamethasone combination when compared with ondansetron alone provides superior control of PONV with delayed PONV being better controlled than early PONV. Both authors compared the ondansetron/dexamethasone combination with 4 mg ondansetron against the 8 mg ondansetron we administered to patients in group B. These studies seem to agree that dexamethasone when combined with either promethazine or ondansetron, may help in reducing the incidence of late PONV.

Nevertheless, the difference in the overall incidence of PONV was statistically significant between the promethazine/dexamethasone and ondansetron groups (*p*=0.022). Meanwhile, Bergess et al. [15] reported that aprepitant, dexamethasone, and promethazine combinations when compared with ondansetron, dexamethasone, and promethazine combinations were similar in efficacy in the prophylaxis of PONV in patients undergoing craniotomy under general anesthesia. Similarly, Habib and coworkers [16] reported that aprepitant/dexamethasone and ondansetron/dexamethasone combinations for prophylaxis of PONV showed no difference in complete response between the groups.

The requirement for rescue antiemetics in this study was lower in patients in the promethazine/dexamethasone group (8.6%) when compared with that in the ondansetron group (20%; *p* > 0.05; Figure 2). Braude and colleagues [30] found no difference in rescue antiemetic use when they compared promethazine with ondansetron, but the incidence of antiemetic use was 18% in the promethazine group and 25% in the ondansetron group. Similarly, Kumar et al. [19] found no difference in the use of rescue antiemetics between ondansetron and dexamethasone prophylaxis but reported incidence in the ondansetron group (25% vs. 20%) and the dexamethasone group (20% vs. 8.6%) was higher. The authors [19] also used a lower dose of ondansetron (4 mg vs. 8 mg), and dexamethasone monotherapy, unlike our promethazine/dexamethasone combination.

The comparative incidence of intraoperative nausea and vomiting obtained in the index study was, however, relatively low and was similar in both study groups. Those patients who required rescue antiemetics in both groups were observed to have intraoperative nausea and vomiting within the first 30 min of the procedure. The requirement for a rescue antiemetic may probably be due to the delayed onset of action of the antiemetics prophylaxis that was administered. The onset of action of promethazine and ondansetron is 30 min for each while dexamethasone is 1 h, as most of the patients were observed to have nausea and vomiting within 30 min postinduction of anesthesia.

This study showed comparable hemodynamic parameters and few side effects that were similar in both groups (Table 5; Figures 3 and 4). This is similar to that reported by Bergess et al. [15]and Singer et al. [17] where no adverse events directly related to dexamethasone or ondansetron medications were found. There was no significant difference in the incidence of hypotension, bradycardia, sedation, shivering, pruritus, and backache between the two groups. The incidence of hypotension was 5.7% in each group (Figure 4). However, Demirhen et al. [30] reported an incidence of hypotension of 14% in patients who received ondansetron. The authors [30] conducted their study on patients undergoing caesarean section, unlike our patients who had myomectomy. The risk of hypotension may be higher in patients undergoing caesarean section under spinal anesthesia compared with other surgical procedures due to the physiologic changes in the spinal space in pregnancy. Bradycardia was managed with 0.01 mg/kg of atropine.

The treatment of hypotension (BP < 90/60 mmHg) in spinal anesthesia involves crystalloids or colloids and vasopressors such as ephedrine and phenylephrine. [31, 32] Ephedrine has been reported to possess additional antiemetics properties, particularly in association with hypotension following spinal anesthesia. [33]

Few patients were observed to have sedation in the promethazine/dexamethasone group and ondansetron group, respectively, but the difference was not statistically significant (Table 5). However, Olatosi et al. [34] found in their study that postoperative drowsiness was significantly prominent with the use of promethazine compared with ondansetron. Even though the authors [34] enrolled only female patients, the procedures were done under general anesthesia with a synergistic effect of promethazine in contrast to our study done under spinal anesthesia. It has been suggested that the sedative effect of promethazine might be dose-dependent in another study. [35] There was no difference in sedation between 6.25 mg, 12.5 mg, and 25 mg doses. However, in the study by Talebpour et al., [11] the higher dose of promethazine used (50 mg) caused the patients to be more sedated, hence it can be suggested that the dose of promethazine for prophylaxis against PONV should not exceed 25 mg. Patients were categorized into sedated and not sedated using Ramsay's sedation score [36]: patients were regarded to have been sedated when the score is greater than three and not sedated when the score is less than three.

Shivering occurred in 12 patients in this study. The difference was not statistically significant and since it occurred comparably in both groups, the study drugs may not be contributory. The low incidence of shivering seen in the index study may probably be due to the addition of fentanyl to bupivacaine in the establishment of the subarachnoid block in both study groups as it has been shown that fentanyl can be used for the treatment of shivering following subarachnoid block. [37]

Backache was also observed. This was comparable in patients in the promethazine/dexamethasone group and the ondansetron group, respectively, the difference was not statistically significant (*p*=1.000). The backache may probably be due to surgical positioning. However, the symptom subsided and then later resolved with the administration of an analgesic postoperative pain management.

Pruritus was observed in the ondansetron group, but no patient in the promethazine/dexamethasone group had pruritus. Pruritus may be related to the opioid (fentanyl) that was used in addition to bupivacaine in both groups, as opioids are known to cause histamine release. However, the absence of pruritus in the promethazine/dexamethasone group is attributable to the antihistamine promethazine.

There was no statistically significant difference in the incidence of respiratory depression in both groups as respiratory depression occurred in only one patient in the ondansetron group, and this may be as a result of the opioid that was used.

Earlier studies showed that patients in both groups expressed a high rate of satisfaction with prophylaxis of PONV (85.8% vs. 80.0%), and there was no significant difference observed between the two groups (*p*=0.705). However, findings in the present study support the efficacy of a combination of low-dose promethazine and dexamethasone as prophylactic antiemetics for the management of postoperative nausea and vomiting following spinal anesthesia.

Combination of low-dose promethazine and dexamethasone is as effective as ondansetron in the prophylaxis of PONV in patients undergoing myomectomy under spinal anesthesia. Both groups were statistically comparable in terms of antiemetic requirements, hemodynamic variability, and side effects profile. Patients who received a combination of promethazine and dexamethasone exhibited a higher rate of satisfaction compared with those given ondansetron. As a result of the easy availability of promethazine and dexamethasone, it is encouraged that a combination of these drugs is effective in the prevention of PONV in patients undergoing myomectomy under spinal anesthesia.

However, the limitation of this study is the absence of a control group (placebo), as it will be unethical to deprive a patient with a high risk for postoperative nausea and vomiting of antiemetic prophylaxis. There was no significant difference in the patient satisfaction profile in both groups (Table 5).

## 5. Conclusion

There was no statistical difference in the incidence of PONV, hemodynamics, and patient satisfaction in both groups. The use of monotherapy as prophylaxis for PONV has not yielded a better outcome when compared with the combination therapy approach. It will be relevant to note that PONV can be relieved effectively to achieve early recovery and ambulation, short hospital stay, reduced cost of care, and above all, improved patient's satisfaction.

The burden of PONV following myomectomy under spinal anesthesia is still a challenge in many centers, especially in developing countries where resources are limited. It is therefore recommended that a combination of low-dose promethazine and dexamethasone, is a cost-effective alternative to ondansetron in prophylaxis of PONV with insignificant side effects; a benefit for modern health care policies where cost reduction is a point of emphasis.

## Figures and Tables

**Table 1:** Demographic and clinical characteristics of patients in both study groups. data presented in mean ± SD.

Variables	Group A	Group B	Total	*p*-value	Sig.
	*N* = 35	*N* = 35			
Age (years)	42.00 ± 10.25	43.65 ± 11.40	—	0.532	NS
Weight (kg)	67.85 ± 10.77	65.82 ± 6.14	—	0.336	NS
Height (m)	1.59 ± 0.07	1.58 ± 0.10	—	0.738	NS
BMI (kg/m^2^)	26.34 ± 2.88	25.52 ± 2.81	—	0.235	NS

ASA (*n*/%)					
1	29 (82.9)	33 (94.3)	62 (88.6)		NS
2	6 (17.1)	2 (5.7)	8 (11.4)		

NS – not significant.

**Table 2:** Baseline vital signs in both study groups.

Variables	Group A	Group B	*p*-value	Sig.
	*N* = 35	*N* = 35		
	Mean ± SD	Mean ± SD		
PR	85.11 ± 9.69	81.54 ± 14.86	0.238	NS
SBP	124.60 ± 6.26	126.34 ± 8.16	0.320	NS
DBP	79.20 ± 7.23	77.43 ± 5.98	0.268	NS
MAP	94.11 ± 9.19	90.54 ± 6.50	0.065	NS
RR	18.34 ± 1.71	18.06 ± 1.78	0.496	NS
SpO_2_	99.57 ± 0.74	99.69 ± 0.63	0.489	NS

NS – not significant.

**Table 3:** Incidence of early and late PONV in both study groups.

PONV		Group A	Group B	Total	*p*-value	Sig.
		*N* = 35	*N* = 35			
		Number (%)	Number (%)			
Early	30 min	2 (5.7)	6 (17.1)	8 (11.4)	0.259	NS
	1 hr	1 (2.9)	1 (2.9)	2 (2.9)	0.368	NS
	2 hr	2 (5.7)	2 (5.7)	4 (5.7)	0.513	NS
	3 hr	0 (0.0)	2 (5.7)	2 (2.9)	0.493	NS
	Total	5 (14.3)	11 (31.4)	16 (45.7)	0.149	NS

Late	4 hr	1 (2.9)	4 (11.5)	5 (7.1)	0.356	NS
	6 hr	1 (2.9)	2 (5.7)	3 (4.3)	1.000	NS
	12 hr	0 (0.0)	0 (0.0)	0 (0.0)	1.000	NS
	24 hr	0 (2.9)	0 (0.0)	0 (0.0)	1.000	NS
	Total	2 (5.7)	6 (17.1)	8 (11.4)	0.259	NS
	Overall	7 (20)	17 (48.6)	24 (34.3)	0.022	S

NS – not significant; S – significant.

**Table 4:** Subarachnoid block characteristics in both study groups.

Variables		Group A	Group B	Total	*p*-value	Sig.
		*N* = 35	*N* = 35			
		Number (%)	Number (%)			
Motor block	Bromage 3	3 (8.6)	5 (14.3)	8 (11.4)	0.355	NS
	Bromage 4	32 (91.4)	30 (85.7)	62 (88.6)		

Sensory block (dermatome)	T4	8 (22.9)	10 (28.6)	18 (25.7)	0.641	NS
	T5	4 (11.4)	2 (5.7)	6 (8.6)		
	T6	23 (65.75)	23 (65.7)	46 (65.7)		

NS – not significant.

**Table 5:** Intraoperative and postoperative complications and side effects.

Variables	Group A *N* = 35	Group B *N* = 35	Total	*p*-value	Sig.
	Number (%)	Number (%)			
Sedation (score > 3)	7 (20.0)	4 (11.4)	11 (15.7)	0.324	NS
Shivering	7 (20.0)	5 (14.3)	12 (17.1)	0.752	NS
Bradycardia	4 (11.4)	1 (2.9)	5 (7.1)	0.325	NS
Hypotension	2 (5.7)	2 (5.7)	4 (5.7)	1.000	NS
Backache	2 (5.7)	1 (2.9)	3 (4.3)	1.000	NS
Pruritus	0 (0.0)	1 (2.9)	1 (1.4)	1.000	NS
Respiratory depression	0 (0.0)	1 (2.9)	1 (1.4)	1.000	NS

NS – not significant.

## Data Availability

The data used to support the findings of the study can be obtained from the corresponding author upon request.
